# Inhibition of the spinal astrocytic JNK/MCP-1 pathway activation correlates with the analgesic effects of tanshinone IIA sulfonate in neuropathic pain

**DOI:** 10.1186/s12974-015-0279-7

**Published:** 2015-03-25

**Authors:** Jun Tang, Chao Zhu, Zhi-hong Li, Xiao-yu Liu, Shu-kai Sun, Ting Zhang, Zhuo-jing Luo, Hui Zhang, Wei-yan Li

**Affiliations:** Department of Anesthesiology, Jinling Hospital, Medical School of Nanjing University, No. 305 East Zhongshan Road, Nanjing, 210002 People’s Republic of China; Department of Anesthesiology, School of Stomatology, The Fourth Military Medical University, No. 145 West Changle Road, Xi’an, 710032 People’s Republic of China; Institute of Orthopaedics, Xijing Hospital, The Fourth Military Medical University, No. 15 West Changle Road, Xi’an, 710032 People’s Republic of China; Department of Neurosurgery, Tangdu Hospital, The Fourth Military Medical University, No. 1 Xinsi Road, Xi’an, 710038 People’s Republic of China; Department of Anatomy, Histology and Embryology, K. K. Leung Brain Research Centre, The Fourth Military Medical University, No. 169 West Changle Road, Xi’an, 710032 People’s Republic of China

**Keywords:** Astrocytes, MCP-1, Neuropathic pain, Tanshinone IIA sulfonate, Analgesic

## Abstract

**Background:**

Neuropathic pain (NP) continues to be challenging to treat due to lack of effective drugs. Accumulating evidence elucidated that glia-mediated inflammatory reactions play a pivotal role in the introduction and development of NP. Besides, activation of the c-Jun N-terminal kinase (JNK)/monocyte chemoattractant protein-1 (MCP-1) pathway in astrocytes has been reported to be critical for spinal astrocytic activation and neuropathic pain development after spinal nerve ligation (SNL). Tanshinone IIA, a major active component of a traditional Chinese drug, Danshen, possesses potent immuno-suppressive activities. The present study was undertaken to assess whether intraperitoneal administration of tanshinone IIA sulfonate (TIIAS) has analgesic effect on SNL-induced neuropathic pain and whether the inhibition of astrocytic activation and JNK/MCP-1 pathway is involved in the analgesic effect of TIIAS.

**Methods:**

The effects of TIIAS on SNL-induced mechanical allodynia were assessed by behavioral testing. Immunofluorescence histochemical staining was used to detect changes of spinal astrocytes and spinal pJNK expression and localization. Immunofluorescence histochemistry and Western blot analysis were used to quantify the SNL-induced spinal pJNK expression after TIIAS administration. Enzyme-linked immunosorbent assay (ELISA) was used to detect the SNL-induced spinal expression of pro-inflammatory cytokines and MCP-1.

**Results:**

Our results indicated that intraperitoneal TIIAS up-regulated the mechanical paw withdrawal threshold (PWT) of NP, while astrocytic activation was suppressed and accompanied by the down-regulation of IL-1β and TNF-α expression, as well as JNK phosphorylation in the spinal dorsal horn. Additionally, the release of MCP-1 was dose dependently decreased. After co-treatment with TIIAS and JNK inhibitor (SP600125), no significant increases in mechanical PWT and MCP-1 expression were observed compared with the TIIAS-treated group.

**Conclusions:**

The present results suggest that the analgesic effects of TIIAS in neuropathic pain are mainly mediated by the down-regulation of SNL-induced astrocytic activation, which is via the inhibition of JNK/MCP-1 pathway.

## Background

Neuropathic pain (NP) is a devastating disease that affects millions of people worldwide [1-4]. Many drugs have been applied in the clinic such as tricyclic anti-depressants, anti-epileptics, and opioids but fail to provide adequately pain relief accompany with unacceptable side effects [3-5]. Hence, it is important to find alternative therapies for the treatment of NP. However, the cellular and molecular mechanisms underlying NP have yet to be fully elucidated. In recent years, substantial evidence has shown that glial cells play an important role in the generation and maintenance of NP [6-8]. Activated glial cells produce numerous mediators such as pro-inflammatory cytokines and growth factors that enhance neuronal activity. Previous studies have suggested that microglia is critical for the initiation of NP, while astrocytes are mainly responsible for the maintenance of NP. During the early stages of NP, microglia in the spinal cord dorsal horn is activated by the central terminals of primary sensory neurons [9-11]. However, activation of astrocytes is crucial for the maintenance of pain. Recently, it has been demonstrated that c-Jun-N-terminal kinase (JNK), a member of the mitogen-activated protein kinase (MAPK) pathway, is expressed predominately by astrocytes and rapidly phosphorylated once the astrocyte is activated [12,13]. Activated astrocytes then secrete numerous neuromodulators, sensitizing the neuronal cell bodies and may, in the long term, bring about plastic changes [14].

**Figure 1:**
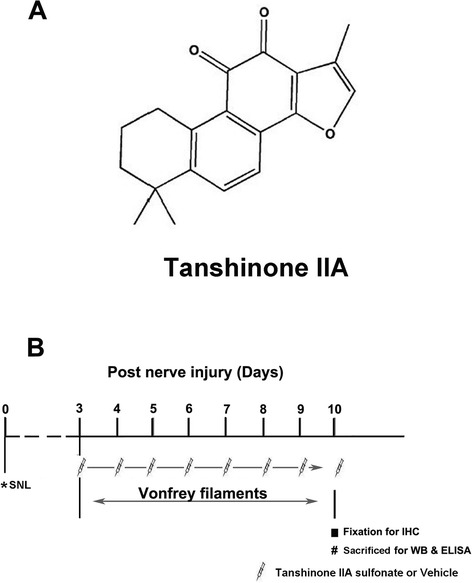
**Chemical structure of tanshinone IIA (TIIA) (A) and detailed timeline of the experiments (B).** POD, post-operative day; SNL, spinal nerve ligation; TIIA, tanshinone IIA; Veh, vehicle; IHC, immunohistochemistry; WB, Western blot; ELISA, enzyme-linked immunosorbent assay.

Danshen, a well-known traditional Chinese herbal medicine derived from the dry roots of *Salvia miltiorrhiza* Bunge, has a long history in treating cardiovascular and cerebrovascular diseases in China [15,16]. Tanshinone IIA (TIIA) (chemical structure shown in Figure 1A), one of the major active components of Danshen extracts, has been found to have anti-inflammatory, anti-oxidative, and anti-apoptotic properties [17-19]. Moreover, our previous studies indicated that tanshinone IIA sulfonate (TIIAS) administration alleviated complete Freund’s adjuvant (CFA)-induced inflammation and inflammatory pain [20], suggesting that TIIAS is a promising anti-inflammatory and anti-nociceptive drug. However, whether TIIAS produces protective effects to alleviate NP is not clear yet, and the underlying mechanisms of anti-nociceptive effects of TIIAS on NP are also need to be explored.

In the present study, utilizing the spinal nerve ligation (SNL) pain model, we evaluated the possible anti-nociceptive effects of TIIAS and its effects on astrocytic activation. Considering the evidence for the inhibitory effects of TIIAS on inflammatory action and the JNK pathway, we also investigated the expressions of several pro-inflammatory cytokines as well as monocyte chemoattractant protein-1 (MCP-1) after TIIAS administration. Specific JNK inhibitor was also administrated to clarify the possible mechanisms underlying the anti-nociceptive effects of TIIAS.

## Methods

### Animal and experimental design

Male Sprague–Dawley rats (220 to 250 g) were housed in a temperature-controlled room in plastic cages (6 animals per cage) with free access to food and water at 22°C to 25°C on a 12-h light/dark cycle. All of the experiments reported in this study were conducted according to an experimental protocol approved by the Animal Use and Care Committee for Research and Education of The Fourth Military Medical University (Xi’an, China). All efforts were made to minimize the animals’ suffering and the number of animals used [21].

The experimental design was as follows: Firstly, to verify the analgesic effects of TIIAS in neuropathic pain and explore the related mechanisms, 144 rats were divided into 6 groups in the experiments: sham-vehicle group, sham-TIIAS (50 mg/kg) group, sham-vehicle group, SNL-TIIAS (2 mg/kg) group, SNL-TIIAS (10 mg/kg) group, and SNL-TIIAS (50 mg/kg) group (*n* = 24 in each group). In each group, 12 rats were used for the tests of mechanical hypersensitivity and the other 12 rats were used for immunofluorescence, Western blot, and enzyme-linked immunosorbent assay (ELISA), 4 rats respectively. Secondly, to assess whether the intraperitoneal administration of TIIAS influenced motor function, another 24 rats were divided into 4 groups for rotarod test: naïve group, TIIAS (2 mg/kg) group, TIIAS (10 mg/kg) group, and TIIAS (50 mg/kg) group (*n* = 6 in each group). Thirdly, to further verify the role of JNK in the analgesic effects of TIIAS, specific JNK inhibitor (SP600125) was administrated and another 80 rats were divided into 5 groups: sham group, SNL-vehicle group, SNL-TIIAS (ED_50_) group, SNL-SP600125 group, and SNL-TIIAS (ED_50_)-SP600125 group (*n* = 16 in each group). In each group, 12 rats were used for the test of mechanical hypersensitivity and the other 4 rats were used for ELISA test of MCP-1 expression on post-operative day (POD) 10 of SNL injury.

### Spinal nerve ligation

SNL was performed according to our previous protocols [22,23]. Briefly, rats were anesthetised with chloral hydrate (300 mg/kg, i.p.). A midline incision was then made at the L3 to S2 level, and the dorsal vertebral column from L4 to S2 was exposed. After a portion of the L6 transverse process was carefully removed, the left L5 spinal nerve was carefully isolated and tightly ligated distal to the dorsal root ganglion (DRG) with 6–0 silk thread. Sham-operated animals were subjected to a similar surgical procedure in which the isolated spinal nerves were not ligated.

### Drug administration

TIIAS (99% purity) was purchased from the National Institute for the Control of Pharmaceutical and Biological Products (NICPBP; Beijing, China). TIIAS was dissolved in physiological saline for the *in vivo* experiments. And the doses for the intraperitoneal administration of TIIAS (three different doses: 2, 10, and 50 mg/kg, i.p.) were selected according to previous studies [19,20]. Considering that astrocytes are activated from POD 3 of SNL, TIIAS or saline was administrated intraperitoneally 30 min prior to behavioral experiments from POD 3 to POD 10 once a day. To further verify the role of JNK in the analgesic effects of TIIAS, a specific JNK inhibitor SP600125 (5 μg/rat, in 5 ul normal saline containing 1% DMSO) or vehicle (normal saline containing 1% DMSO) was intrathecally administrated 15 min prior to the measurement of mechanical pain behaviors from POD 3 to POD 10 after SNL. Intrathecal implantation was performed by inserting polyethylene (PE) tubing to inject the drug directly into the subarachnoid space of the lumbar enlargement. After surgery, the neurologically normal rats were injected with 2% lidocaine (10 μl) through the intrathecal catheter to confirm whether the PE tubing was in the intrathecal space. Only the rats showing complete paralysis of both hind legs and tail after administration of lidocaine were used for the subsequent experiments. At the end of each experiment, the position of the PE tubing in the intrathecal space at the lumbar enlargement was verified visually by exposing the lumbar spinal cord. Data from rats with incorrect PE tubing position were abandoned from the study.

### Rotarod testing

Motor dysfunction can have evident effects on nociceptive behavioral tests [24]. To assess whether the intraperitoneal administration of TIIAS influenced motor function, another 24 rats were used in rotarod tests via an accelerating rotating rod (Ugo Basile 7650, Varese, Italy). After 1 min of training, rats were placed on the rotarod, which was linearly accelerated from 4 to 40 rpm over 5 min. The elapsed time before the rat fell on each of the three runs with 10-min intervals between runs was recorded for each rat. The test was repeated 30 min after the intraperitoneal administration of TIIAS or saline once per day for 7 days, and the time that the rat remained on the rotarod was recorded. Final results are expressed as a percentage of baseline value of each rat.

### Mechanical hypersensitivity

Rats were placed on an elevated mesh grid that completely exposed the middle of the hind paw. Mechanical hypersensitivity was tested using von Frey filaments (Stoelting, Kiel, WI, USA) by experimenters who were blinded to group assignment as described previously [25]. The stiffness of the von Frey filaments was 2, 4, 6, 8, 10, 15, and 26 g. The hind paw was pressed with each filament, in order of increasing stiffness, for 5 s. Rapid pulling back, biting, or shaking of the hind limb within 5 s of the hind limb being pricked by one of the von Frey filaments was taken as a positive sign of withdrawal. The interval between trials was at least 5 min. For each trial, the same hind limb was stimulated ten times by a single von Frey filament before being stimulated by the next larger filament. The minimal value that resulted in at least six responses to ten stimulations was recorded as the paw withdrawal thresholds (PWTs). The formula for calculating the percentage change was 100–100 × (baseline of SNL-TIIAS − post-SNL-TIIAS)/(baseline of SNL-vehicle − post-SNL-TIIAS).

### Immunofluorescence

The rats were deeply anesthetised by injection of pentobarbital (60 mg/kg, i.p.) and transcardially perfused with 200 ml of 5 mM sodium phosphate-buffered 0.9% (*w*/*v*) saline (PBS; pH 7.3), followed by 500 ml of 4% (*w*/*v*) paraformaldehyde in 0.1 M phosphate buffer (PB; pH 7.4). The L5 spinal cord segments were harvested and immersed in 30% (*w*/*v*) sucrose in 0.1 M PB overnight at 4°C. Transverse spinal sections (25-μm thickness) were then cut on a cryostat (Leica CM1800; Heidelberg, Germany). After been rinsed in PBS (pH 7.2 to 7.4) 3 times (10 min each), the sections were incubated for 2 h at room temperature and then for 48 h at 4°C with anti-glial fibrillary acidic protein (GFAP) mouse IgG (1:5,000; Chemicon, Temecula, CA, USA) or anti-GFAP mouse IgG (1:5,000; Millipore, Billerica, MA, USA) mixed with anti-pJNK rabbit IgG (1:100; Cell Signalling, Danvers, MA, USA) in PBS containing 0.3% (*v*/*v*) Triton X-100, 0.25% (*w*/*v*) *λ*-carrageenan and 5% (*v*/*v*) donkey serum (PBS-XCD). After being washed three times in 0.01 M PBS (10 min each), the sections were incubated for 4 h at room temperature with Alexa 594-conjugated donkey anti-mouse IgG (1:500; Millipore, Billerica, MA, USA) or a mixture of Alexa 488-conjugated donkey anti-rabbit IgG and Alexa 594-conjugated donkey anti-mouse IgG. After staining, all of the sections were mounted onto glass slides and cover-slipped with 50% (*v*/*v*) glycerol and 2.5% (*w*/*v*) triethylenediamine (anti-fading agent) in 0.05 M PBS. Using a confocal laser-scanning microscope (FV1000; Olympus, Tokyo, Japan), the sections were observed with the appropriate laser beams and filter settings for green-emitting Alexa 488 (excitation 488 nm; emission 530 nm) or red-emitting Alexa 594 (excitation, 543 nm; emission, 590 to 615 nm). The digital images were captured using FV10-ASW-1.6 software (Olympus, Tokyo, Japan), modified (15% to 20% contrast enhancement) in Photoshop CS4 (Adobe Systems, San Jose, CA, USA) and then saved as TIFF files.

### Western blot analysis

Animals were deeply anesthetised by injection of pentobarbital (60 mg/kg, i.p.) and then rapidly sacrificed. The L5 spinal cord segments were dissected on ice according to the termination of the L4 and L5 dorsal roots. The left dorsal part of spinal cord was further split and then homogenized with a hand-held pestle in sodium dodecyl sulphate (SDS) sample buffer (10 ml/mg tissue) containing a mixture of proteinase and phosphatase inhibitors (Sigma-Aldrich, St. Louis, MO, USA). The protein concentrations were estimated using the bicinchoninic acid (BCA) method. The samples were heated in boiling water for 8 min, loaded onto 10% SDS-polyacrylamide gels (Bio-Rad Laboratories, Hercules, CA, USA) and transferred to polyvinylidene difluoride membranes (PVDF; Immobilon-P, Millipore, Billerica, MA, USA). Membranes were blocked in a 5% BSA solution for 2 h and probed with the following primary antibodies overnight at 4°C: anti-GFAP mouse IgG (1:5,000; Chemicon, Temecula, CA, USA), anti-pJNK rabbit IgG (1:300; Cell Signalling, Danvers, MA, USA), anti-JNK rabbit IgG (1:300; Cell Signalling, Danvers, MA, USA), and anti-β-actin mouse IgG (1:3,000, Sigma-Aldrich, St. Louis, MO, USA). The membranes were then incubated with the following secondary antibodies for 2 h: horseradish peroxidase (HRP)-conjugated donkey anti-rabbit IgG (1:5000; Zhongshan, Beijing, China) or HRP-conjugated donkey anti-mouse IgG (1:5,000; Zhongshan, Beijing, China). The membranes were rinsed three times (10 min each) with Tris-buffered saline with Tween-20 (TBST) between each step. All reactions were detected by the enhanced chemiluminescence (ECL) detection method (Amersham Corporation, Arlington Heights, IL, USA). Restore Western blot stripping buffer (Pierce Biotechnology, Inc., Rockford, IL, USA) was used to detect the different proteins with close molecular weight in the same PVDF membranes. Briefly, after the antibodies for detecting pJNK were stripped, the same PVDF membrane was used for the detection of β-actin. The detailed steps were carried out according to the manufacturer’s instructions. The densities of protein blots were analyzed using Labworks Software (Ultra-Violet Products, Cambridge, UK). The densities of target proteins and β-actin immunoreactive bands were quantified with background subtraction. The same size of square was drawn around each band to measure the density, and the background near that band was subtracted. Target protein levels were normalized against β-actin levels and expressed as relative fold changes compared to the sham-vehicle group. The intensity of blots was quantified with densitometry by the persons who were blinded to the treatments.

### Enzyme-linked immunosorbent assay

The left dorsal horns of the L5 spinal segments of animals in different groups were split according to the same method used for Western blot analysis. Spinal cord tissues were homogenized in a lysis buffer containing protease and phosphatase inhibitors. The amounts of IL-6, TNF-α, IL-1β, and MCP-1 were measured by enzyme-linked immunosorbent assays using corresponding ELISA kits (R&D Systems, Minneapolis, MN, USA).

### Quantification and statistical analysis

#### Statistical analysis

All data were collected and analyzed by researchers blinded to the surgery and reagents that were used. Repeated measures of ANOVA (with Bonferroni confidence interval adjustment) tests were conducted for the data from the rotarod and von Frey experiments. Data from the Western blot and ELISA tests were analyzed using a one-way ANOVA followed by least significant difference (LSD) for *post hoc* analysis. All of the data are presented as mean ± SEM, and all statistical analysis was performed using SPSS software version 16.0 (SPSS Inc., Chicago, IL, USA). A *P* value of <0.05 was considered statistically significant.

### Dose-effect curve and ED_50_ calculation

The TIIAS dosages were transformed into logarithm dose with Prism, and the non-line fit was performed so as to build the dose-effect curve. Based on the dose-effect curve, the ED_50_ of the effects of TIIAS on analgesia was calculated. The reliability of the ED_50_ calculated from a specific dose-effect curve was evaluated using the slope factor generated by the GraphPad Prism version 5.01 for Windows (San Diego, CA, USA, www.graphpad.com).

## Results

### Effects of intraperitoneal administration of TIIAS on rotarod test

**Figure 2:**
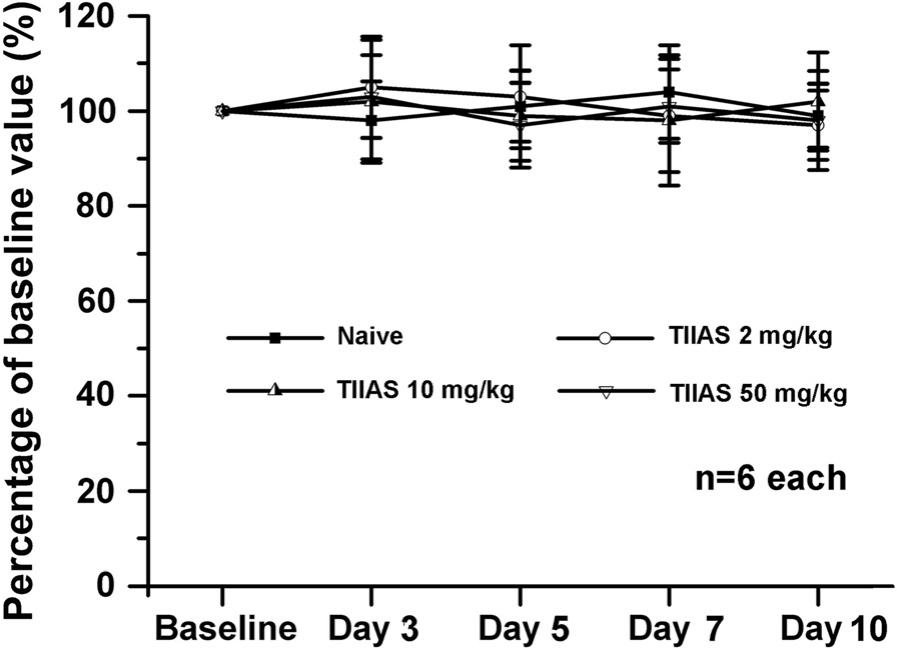
**The effects of intraperitoneal administered tanshinone IIA sulfonate (TIIAS) on motor performance of normal rats in the rotarod test.** Compared with the baseline response, intraperitoneal administration of TIIAS (2, 10, or 50 mg/kg) for 10 days did not affect motor performance. The results were expressed as a percentage of each rat’s own baseline value. TIIAS, tanshinone IIA sulfonate.

Motor dysfunction can have evident effects on nociceptive behavioral results, and as such, it is essential to assess whether the dosages of TIIAS in the present study (2, 10, and 50 mg/kg) could induce impairment of motor functions. To determine this, a rotarod test was carried out to assess the influence of TIIAS administration on motor function. As shown in Figure 2, there was no difference in the performance of the rats in the vehicle control group or in the TIIAS treatment (2, 10, and 50 mg/kg) group, indicating that intraperitoneal administration of TIIAS did not have an obvious measurable effect on motor functions.

### Intraperitoneal administration of TIIAS attenuated SNL-induced mechanical hypersensitivity in a dose-dependent manner

**Figure 3:**
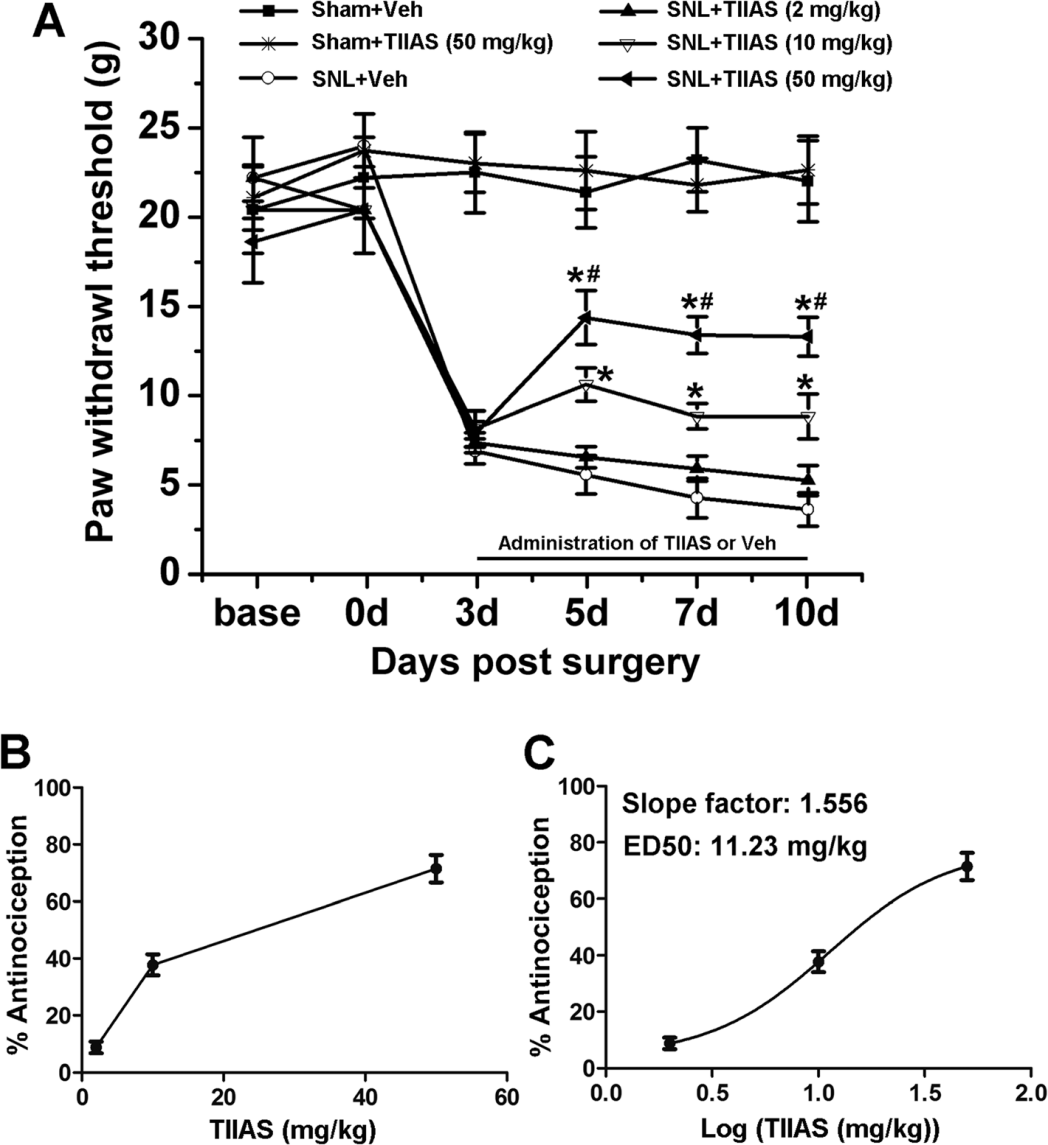
**The effects of intraperitoneal TIIAS on the SNL-induced mechanical allodynia.** SNL injury resulted in prominent mechanical allodynia. Intraperitoneal injection of TIIAS did not affect the pain threshold of the sham-operated group. Intraperitoneal administration of TIIAS (10 or 50 mg/kg, from days 3 to 10, once a day) significantly reduced SNL-induced mechanical allodynia in a dose-dependent manner, whereas 2 mg/kg TIIAS did not change SNL-induced mechanical allodynia **(A)**. *P < 0.05, compared with that of the SNL-vehicle group. #*P* < 0.05, compared with that of the SNL-TIIAS (10 mg/kg) group. The dose-effect or log (dose)-effect curves for analgesic effects of TIIAS were shown in **(B)** and **(C)**, *n* = 12 for each group. SNL, spinal nerve ligation; TIIAS, tanshinone IIA sulfonate; Veh, vehicle.

Next, we assessed whether TIIAS had effects on preventing mechanical hypersensitivity in the neuropathic pain model of SNL. SNL injury resulted in prominent mechanical hypersensitivity as demonstrated in the SNL-vehicle group (Figure 3A). Compared with that of the SNL-vehicle group, intraperitoneal injection of 2 mg/kg TIIAS did not influence PWT. However, TIIAS (10 and 50 mg/kg) treatment prevented SNL-induced mechanical allodynia of ipsilateral hind paws from POD 3 to POD 10 (*P* < 0.05, compared with that of the SNL-vehicle group, respectively; Figure 3A). Furthermore, significant differences in PWT were found between the two groups treated with different dosages. TIIAS treatment did not change the basal threshold in the sham-operated group (Figure 3A). These results showed that the anti-allodynia effects of TIIAS appeared to be dose-related. Moreover, the effect of TIIAS on SNL-induced mechanical allodynia was calculated based on the log (dose)-response curve (Figure 3B). To calculate the slope factor and ED_50_, we also calculated from the dose–response curve (Figure 3C). The ED_50_ of TIIAS on SNL-induced mechanical allodynia was 11.23 mg/kg, and the slope factor was 1.556, suggesting that our regimen for dosage selection was robust.

### TIIAS inhibits SNL-induced elevated astrocytic activation

**Figure 4:**
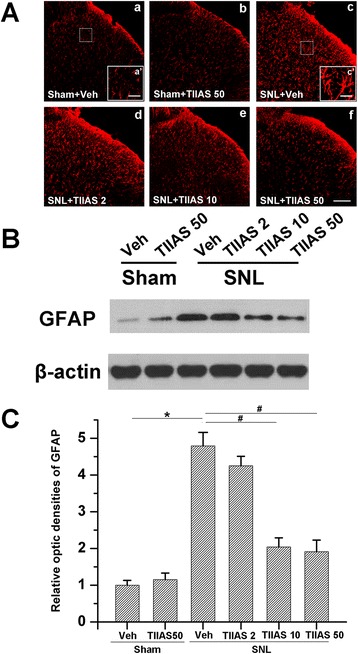
**The effects of intraperitoneal injection of TIIAS on SNL-induced spinal astrocytic activation and glial fibrillary acidic protein (GFAP) expression. (A)** SNL induced a remarkable astrocytic activation indicated by GFAP up-regulation in the ipsilateral spinal dorsal horn. The cell bodies of activated astrocytes appeared hypertrophied, and the processes were thickened. Intraperitoneal TIIAS administration inhibited the immunodensities of GFAP in the ipsilateral spinal dorsal horn after SNL. Scale bar for a-f, 100 μm; scale bar (a’ and c’), 20 μm. **(B)** In accordance with the immunofluorescence results, the Western blot analysis of GFAP showed that SNL-induced GFAP up-regulation was suppressed by intraperitoneal administration of TIIAS in a dose-dependent manner. **(C)** Statistical analysis of the GFAP expressions after different treatments. Asterisk or number sign each indicates statistically significant difference with *P* < 0.05 between groups, *n* = 4 for each group. SNL, spinal nerve ligation; TIIAS, tanshinone IIA sulfonate; Veh, vehicle; GFAP, glial fibrillary acidic protein.

In order to verify whether the anti-allodynic and anti-hyperalgesic effects of TIIAS are accompanied with the inhibition of astrocytic activation, we investigated GFAP expression at POD 10 among the groups. Immunohistochemical data showed that SNL injury induced GFAP expression in the ipsilateral spinal dorsal horn on POD 10 compared with sham controls (Figure 4A). The activated astrocytes by SNL injury also revealed hypertrophied cell bodies and thickened processes. SNL-induced GFAP expression was also verified by Western blot (*P* < 0.05, compared with that of the sham-vehicle group; Figure 4B, C). By contrast, intraperitoneal administration of TIIAS resulted in a decrease in GFAP immunofluorescence in the dorsal horn. Astrocytes in the SNL-TIIAS group were not activated, similar to the sham-vehicle group. Western blot results (Figure 4B,C) also verified that the expression of GFAP was significantly attenuated with 10 and 50 mg/kg TIIAS treatment (*P* < 0.05).

### Astrocytic JNK activation was dose dependently decreased by administration of TIIAS

**Figure 5:**
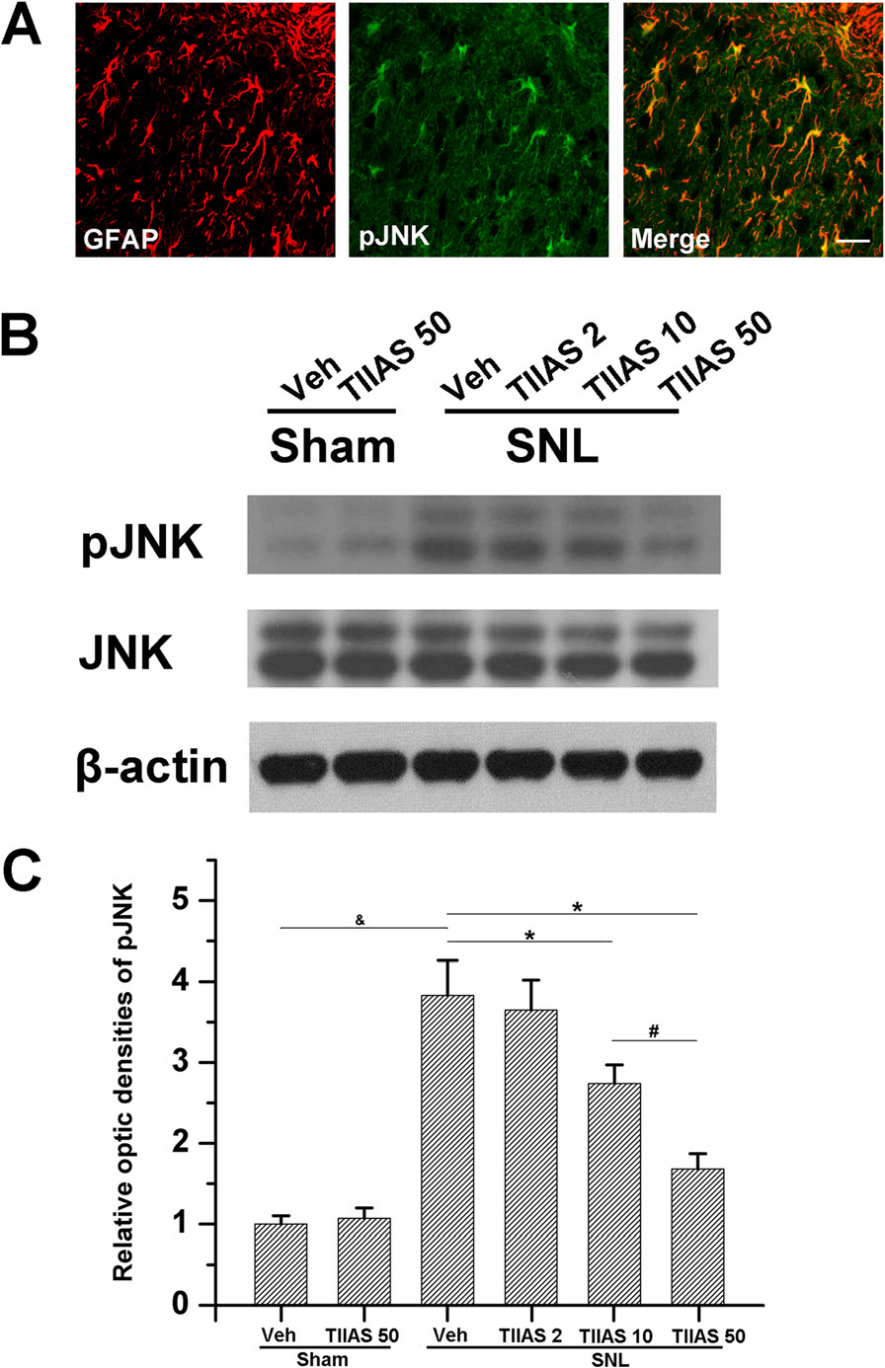
**The effects of intraperitoneal injection of TIIAS on SNL-induced pJNK expression. (A)** High-magnification images demonstrated pJNK (green) was predominantly colocalized with GFAP (red) as shown by overlapped staining (rightmost panel, yellow). Scale bar, 20 μm. **(B, C)** Western blot analysis of pJNK showed that pJNK expression increased in the ipsilateral dorsal spinal cord of SNL rats at POD 10 after surgery. However, SNL-induced pJNK expression was suppressed by intraperitoneal administration of TIIAS in a dose-dependent manner. Asterisk, number sign, or ampersand each indicates statistically significant difference (*P* < 0.05) between groups, *n* = 4 for each group. SNL, spinal nerve ligation; TIIAS, tanshinone IIA sulfonate; Veh, vehicle; JNK, c-Jun N-terminal kinase.

We further investigated the activation of astrocytic JNK after TIIAS treatment. Immunohistochemical data showed that almost all pJNK-positive cells were also GFAP-positive astrocytes (Figure 5A). Additionally, the effect of intraperitoneal administration of TIIAS on JNK phosphorylation was tested. Western blot results showed that after SNL, the expression of pJNK in spinal dorsal horn of the SNL-vehicle group was significantly enhanced compared with that of the sham-vehicle group (*P* < 0.05; Figure 5B,C), while intraperitoneal TIIAS (50 mg/kg, the maximum dose in our studies) did not affect pJNK expression in sham-operated rats compared with that of the sham-vehicle group. Further analysis showed that intraperitoneal administration of TIIAS (2 mg/kg) did not obviously affect pJNK expression compared with the SNL-vehicle group (*P* > 0.05; Figure 5B,C). However, the expression of pJNK was significantly attenuated compared with the SNL-vehicle group by intraperitoneal TIIAS (10 and 50 mg/kg) administration (*P* < 0.05; Figure 5B,C). Additionally, intraperitoneal 50 mg/kg TIIAS showed stronger effects on pJNK expression compared with the SNL-TIIAS (10 mg/kg) group (*P* < 0.05; Figure 5B,C). These results suggest that intraperitoneal TIIAS can effectively inhibit pJNK up-regulation in a dose-dependent manner.

### TIIAS attenuates SNL-induced up-regulation of pro-inflammatory cytokines and MCP-1

**Figure 6:**
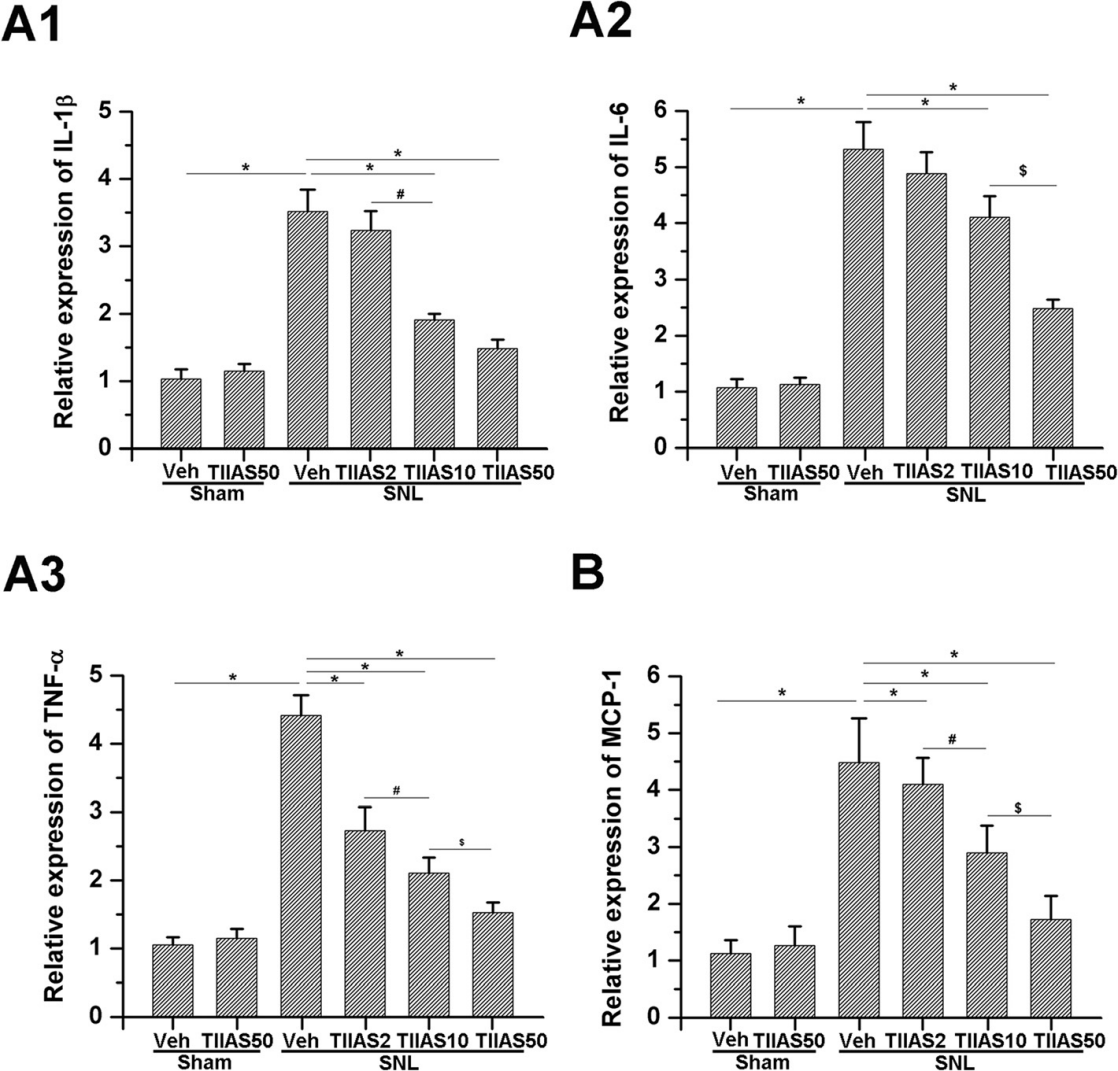
**Effects of intraperitoneal injection of TIIAS on SNL-induced pro-inflammatory cytokines and monocyte chemotactic protein (MCP-1) level in spinal dorsal horn revealed by ELISA.** The levels of IL-1β, IL-6, and TNF-α were increased 10 days after SNL injury in the ipsilateral spinal dorsal horn **(A1-A3, B)**. The lowest dosage (2 mg/kg) of TIIAS did not have any obvious effects on the expression of IL-1β, IL-6, and MCP-1 after SNL injury. However, SNL-induced elevated IL-1β, IL-6, and MCP-1 expression was reduced by the treatment of TIIAS (10 and 50 mg/kg) in a dose-dependent manner. TIIAS (2, 10, and 50 mg/kg) could down-regulate SNL-induced elevated TNF-α in a dose-dependent manner (A1-A3, B). Asterisk, number sign, or dollar sign each indicates statistically significant difference (*P* < 0.05) between groups, *n* = 4 for each group. SNL, spinal nerve ligation; TIIAS, tanshinone IIA sulfonate; Veh, vehicle; IL-1β, interleukin*-*1 beta.

In order to evaluate the possible signal transduction pathways by which TIIAS alleviated SNL-induced hyperalgesia, the effect of intraperitoneal administration of TIIAS on the levels of pro-inflammatory cytokines such as IL-1β, IL-6, and TNF-α as well as the chemokine MCP-1 were investigated. Administration of TIIAS did not change spinal pro-inflammatory cytokine expression in sham-vehicle rats (Figure 6). However, SNL resulted in significantly increased pro-inflammatory cytokine expression in the ipsilateral spinal dorsal horn on POD 10 (*P* < 0.05, respectively, compared with each of the sham-vehicle; Figure 6A1,A2,A3). Our results demonstrated that TIIAS administration inhibited the increased expression of pro-inflammatory cytokines due to SNL. The low dosage of TIIAS (2 mg/kg) could depress TNF-α expression after SNL injury. However, this dosage did not have any obvious effect on the expression of IL-1β and IL-6 after SNL injury. In the higher dosage group (10 and 50 mg/kg), TIIAS could inhibit the increase of these three pro-inflammatory cytokines to varying degrees (*P* < 0.05, compared with the SNL-vehicle group, respectively). Clear differences were observed between the groups with two different dosages (10 and 50 mg/kg) (*P* < 0.05, respectively). Similar results were obtained regarding the role of TIIAS on MCP-1 expression. The low dosage of TIIAS (2 mg/kg) did not have any obvious effect on MCP-1 expression, while higher dosages of TIIAS (10 and 50 mg/kg) resulted in the decrease of MCP-1 (*P* < 0.05, compared with the SNL-vehicle group; Figure 6B). These results suggest that intraperitoneal TIIAS can effectively inhibit SNL-induced up-regulation of pro-inflammatory cytokines and MCP-1 in a dose-dependent manner.

### Analgesic effect of TIIAS via the inhibition of JNK activation in astrocytes after SNL

**Figure 7:**
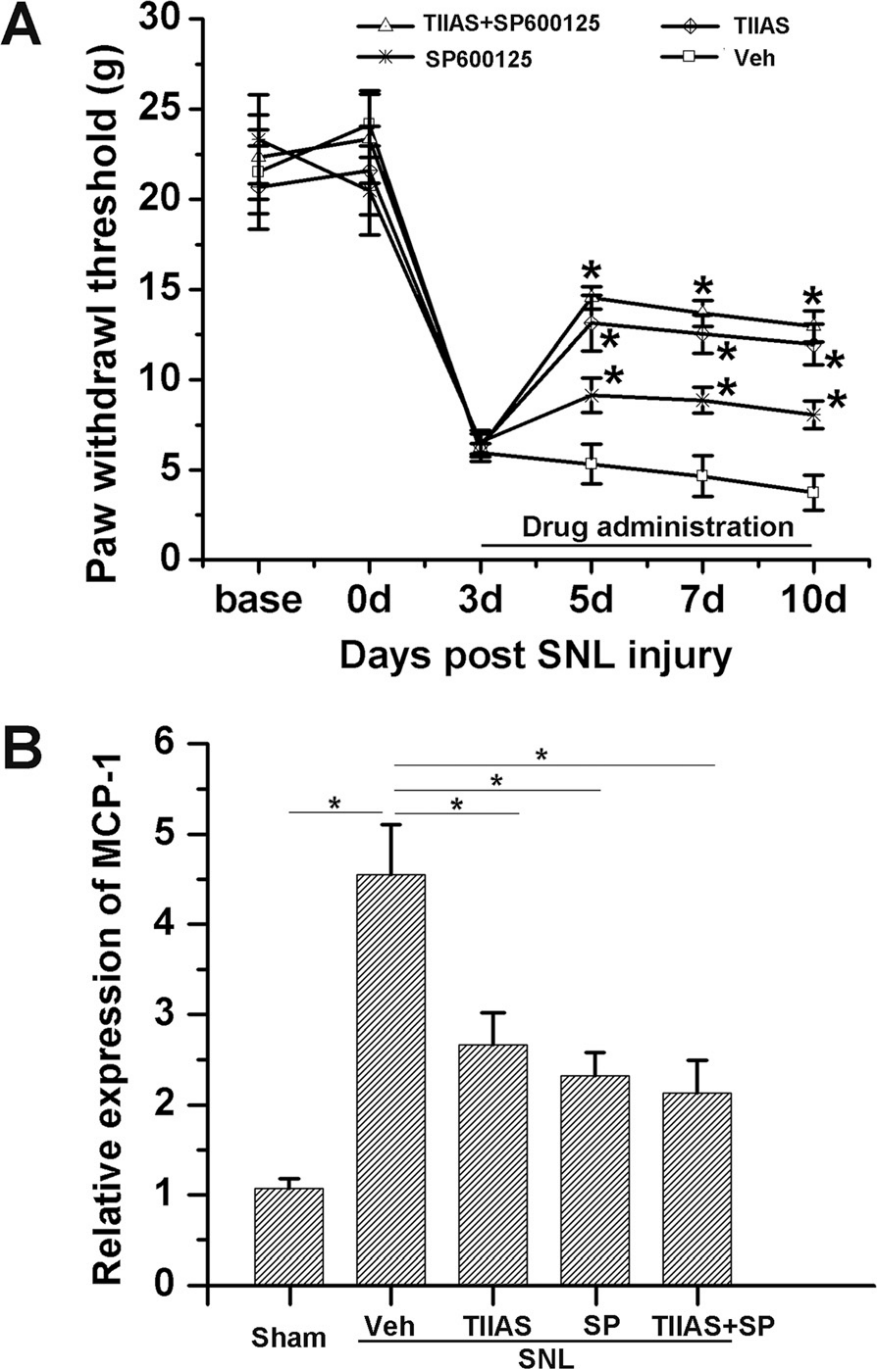
**Analgesic effects and MCP-1 expression with co-treatment of TIIAS and SP600125 in SNL rats.** SP600125, a specific JNK inhibitor, was injected intrathecally at POD 3 to POD 10 (*n* = 12 for each group). Intrathecal administration of SP600125 significantly increased the mechanical PWT compared with the vehicle control, and co-treatment with TIIAS and SP600125 did not lead to more significant increases in mechanical PWT compared with the TIIAS-alone group **(A)**. And also, co-treatment with TIIAS and SP600125 did not result in further inhibition of MCP-1 compared with TIIAS-alone group **(B)**. Data (A) are presented as mean ± SEM (*P < 0.05, *n* = 12, *vs* vehicle group, repeated measures ANOVA). Data (B) are presented as mean ± SD (*P < 0.05, *vs* SNL-vehicle group, *n* = 4). SNL, spinal nerve ligation; TIIAS, tanshinone IIA sulfonate; Veh, vehicle; MCP-1, monocyte chemoattractant protein-1; SP, sodium pyruvate.

To further verify whether the analgesic effects of TIIAS was occurring through the inhibition of JNK activation in astrocytes after SNL, SP600125 (a specific JNK inhibitor), was delivered intrathecally into the L4/5 spinal cord. Intrathecal administration of SP600125 (5 μg) significantly increased the mechanical PWT compared with the vehicle control (*P* < 0.05; Figure 7A). Furthermore, co-treatment with TIIAS and SP600125 did not lead to significant increases in mechanical PWT compared with the TIIAS-alone group (*P* > 0.05; Figure 7A). Additionally, co-treatment with TIIAS and SP600125 did not result in further inhibition of MCP-1 compared with the TIIAS-alone group (*P* > 0.05; Figure 7B). Thus, the analgesic effect of TIIAS appeared to rely mainly on the inhibition of JNK activation.

## Discussion

In the present study, behavioral testing demonstrated that administration of TIIAS alleviated the mechanical allodynia and thermal hypersensitivity of NP rats. Additionally, the activation of astrocytes and the JNK pathway was markedly inhibited, and the production of pro-inflammatory cytokines was also suppressed. We also demonstrated that the anti-nociception effect of TIIAS and JNK inhibitor (SP600125) co-administration on mechanical allodynia was not altered compared with TIIAS administration alone. Furthermore, the expression of MCP-1, which is known to be the downstream chemokine of MAPK pathway, was significantly attenuated by TIIAS administration. Taken together, our results indicate that the analgesic effect of TIIAS is likely mediated wholly or in a large part by inhibiting the activation of JNK/MCP-1 pathway in activated astrocytes after SNL injury.

SNL is a widely used experimental model to stimulate the major events of NP in the rat. In this model, the unilateral L5 spinal nerve was tightly ligated, which results in primary damage of the nerve [26]. This initiates a cascade of inflammatory reactions in the spinal cord, and NP becomes evident. A previous study observed that the mechanical allodynia began to be measureable the third day after SNL and was maintained until the 30th day after primary damage [27]. Danshen, a traditional Chinese drug, has been used in treating several kinds of diseases in China [28]. Tanshinone IIA is one of the major active components of Danshen. Recent studies have reported that TIIAS has potent anti-inflammatory effects [19,29,30]. In the present study, mechanical allodynia began to be obvious from POD 3 after SNL injury, and TIIAS administration significantly alleviated mechanical allodynia from POD 5 to POD 10, indicating that TIIAS may be a beneficial agent for treating NP.

Expression of pro-inflammatory cytokines in the spinal dorsal horn was also down-regulated. Astrocytes play a pivotal role in the introduction and maintenance of NP, acting as the main immune cells in the central nervous system. After the nerve injury, the activation of spinal astrocytes persisted until the 150th day, which was regarded as a main event of the NP-related inflammatory reaction in the spinal dorsal horn [22,31,32]. Recent studies found that L-alpha-aminoadipate, a specific inhibitor of astrocytes, produces dose-dependent inhibition of nerve injury-induced mechanical allodynia [33,34]. In the present study, we also observed the alleviation of mechanical allodynia after the application of TIIAS. Meanwhile, down-regulation of GFAP expression in the spinal dorsal horn was observed, indicating that astrocytic activation was suppressed. These results indicated that the anti-nociceptive effect of TIIAS may possibly be related with the suppression of astrocytic activation.

JNK, a key member of the MAPK pathway, plays an important role in astrocytic activation after peripheral nerve injury [35-37]. The phosphorylated JNK (active form) is increased after SNL, which has been shown to have a critical role in intracellular signal transduction. And intrathecal application of SP600125, a JNK selective inhibitor, either before or after SNL, can increase the mechanical PWT significantly [33,38]. Another JNK inhibitor, D-JNKI-1, also showed potent anti-nociceptive effects on SNL-induced NP [33]. In the present study, SNL-induced increases of pJNK were almost totally blocked by the intrathecal injection of TIIAS, accompanied by the alleviation of tactile allodynia. Furthermore, co-treatment with TIIAS and SP600125 did not result in significant increases in mechanical PWT compared with the TIIAS-alone treatment group. These results indicated that inhibition of JNK in the spinal dorsal horn of SNL rats was consistent with the analgesic effect of TIIAS. Recent studies have indicated that in some pathological processes, activation of JNK can be inhibited by TIIAS, especially in the signal transduction of anti-inflammatory and anti-apoptotic pathways [39-42]. The pro-inflammatory cytokines, especially TNF-α and IL-1β, induced by peripheral nerve injury, are essential triggers for the activation of JNK in spinal astrocytes underlying the development of neuropathic pain. In our study, TIIAS administration also decreased the expression of the three pro-inflammatory cytokines (TNF-α, IL-6, and IL-1β), indicating that the inhibition of the activation of JNK via the suppression of the inflammatory cascade is correlated with the analgesic effect of TIIAS.

Accumulating evidence indicates that MCP-1 is produced by astrocytes via the JNK-mediated pathway after SNL and mediates pain via CCR2 receptors [13,43,44]. Treatment with a JNK inhibitor inhibits production of MCP-1 in IL-1β- or TNF-α-stimulated cultured astrocytes [13,45]. Another study found that propentofylline, an anti-allodynic agent, dampens MCP-1 release from astrocytes [46]. Additionally, mice overexpressing MCP-1 in astrocytes display enhanced nociceptive responses [47]. As for the involvement of MCP-1 in central sensitization, it was also reported that MCP-1 increases pain sensitivity via direct activation of NMDA receptors in dorsal horn neurons [13]. In this study, the analgesic effects of TIIAS were mediated in part by the down-regulation of MCP-1. Increased MCP-1 expression was also reported in DRG neurons after nerve injury [48]. However, in the spinal cord, MCP-1 is predominantly expressed in spinal astrocytes after SNL [13]. In our study, the change of MCP-1 expression in SNL model after TIIAS administration was regarded in the spinal astrocytes.

## Conclusions

**Figure 8:**
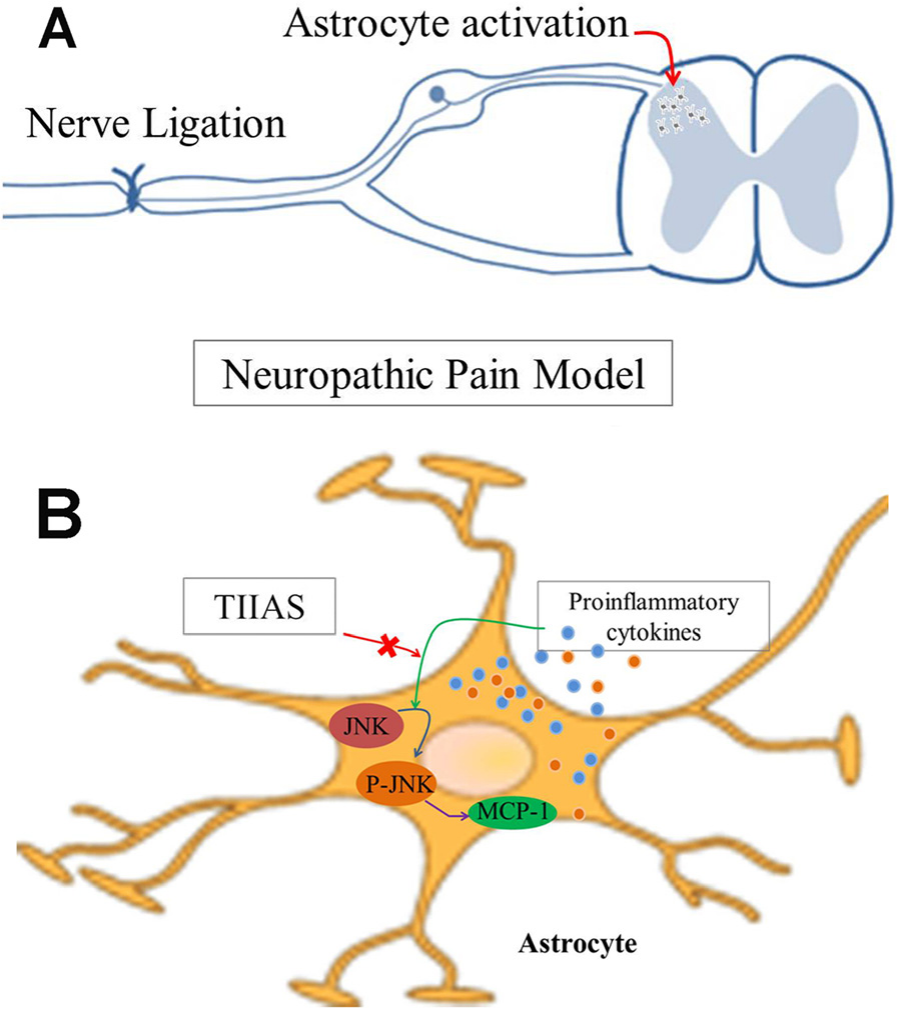
**Schematic illustrating potential mechanisms of the anti-nociceptive effects induced by TIIAS on neuropathic pain**. Peripheral nerve injury (like spinal nerve ligation) increases pro-inflammatory cytokines and induces glial activation in the spinal dorsal horn **(A)**. The potential roles of TIIAS in spinal pain transmission are presented in **(B)**. Peripheral nerve injury induces pro-inflammatory cytokines, causing the activation of spinal JNK pathway in astrocytes. The up-regulation of MCP-1 produced by astrocytes via JNK-mediated pathway after SNL may facilitate the glutamate-related synaptic transmission and enhance neuropathic pain. JNK activation could be the targeting sites of TIIAS. Down-regulation of JNK/MCP-1 may be involved in the anti-nociceptive effect of TIIAS on neuropathic pain. TIIAS, tanshinone IIA sulfonate; JNK, c-Jun N-terminal kinase; MCP-1, monocyte chemoattractant protein-1.

In the present study, we showed that TIIAS had a potent analgesic effect on SNL-induced NP. Our results also indicated that the analgesic effect of TIIAS coincides with the inhibition of the inflammatory reactions in the spinal dorsal horn. Furthermore, the suppression of astrocyte-related JNK phosphorylation and MCP-1 was involved in the analgesic effect of TIIAS (Figure 8). These findings in the present study provide evidence for understanding the mechanisms underlying the anti-nociception effects of TIIAS in a SNL-induced neuropathic pain model and support a novel strategy for treating peripheral nerve injury-induced neuropathic pain.
